# Technique of Arthroscopic Suprapectoral Tenodesis of the Long Head of the Biceps With Interference Screw

**DOI:** 10.1016/j.eats.2021.01.008

**Published:** 2021-03-22

**Authors:** Aleksandr Aleksandrovich Vetoshkin, Hayk Hamlet Aghamalyan, Maksat Khemrakulievich Gurbannazarov

**Affiliations:** a Traumatology and Orthopedics Department, Nikiforov Russian Center of Emergency and Radiation Medicine, EMERCOM of Russia, St. Petersburg, Russia; b Department of Sports Traumatology and Arthroscopic Surgery, University Hospital after A. Miqayelyan, Yerevan, Armenia; c Traumatology and Orthopedics Department, Pavlov First Saint Petersburg State Medical University, St. Petersburg, Russia

## Abstract

Tenodesis and tenotomy are the main surgical options to treat different pathologies of the long head of the biceps tendon. Maintaining the functionality of the tendon during tenodesis makes it more preferable surgical option. The consensus on the most advanced tenodesis technique has not been reached. The article presents the arthroscopic all-inside technique of suprapectoral tenodesis of the biceps tendon using the technique of “zone marking” with 2 spinal needles or pins.

Tenodesis of the long head of the biceps tendon (LHBT) has several advantages compared with tenotomy: (1) LHBT fixation maintains its length and tension, thereby preventing muscle atrophy; (2) it helps to stabilize the head of the humerus^1^; (3) preserves the main functions of the elbow joint—the strength of flexion and supination; and (4) helps to avoid of spastic pain and minimizes cosmetic deformation.^2^, ^3^, ^4^ Taylor et al.^5^ examined the anatomy of the bicipital groove in detail and identified 3 zones. The most proximal zone I is the bony bicipital groove itself, which is surrounded by the synovial membrane and bounded by the fibers of the subscapularis tendon. Zone II is proximally limited by the lower border of the tendon of the subscapularis muscle and the transverse ligament of the brachial bone, and distally by the upper border of the tendon of the pectoralis major muscle. Zone III runs deep under the tendon of the pectoralis major muscle.

Currently, both open and arthroscopic approaches and techniques are used to perform tenodesis. With open tenodesis, fixation can be achieved using an interference screw, bicortical or single-cortical buttons, suture anchor, or suturing using the surrounding soft tissues (suturing of the biceps tendon with rotator cuff or with pectoralis major tendon).^6^^,^^7^ However, open methods have a number of complications, the most important of which is the formation of adhesions around the nerves, which leads to disruption of muscle innervation and loss of sensation.^8^^,^^9^ The formation of a scar on the skin also can be attributed to the negative factors of open tenodesis, which of course reduces the cosmetic result.

Arthroscopic techniques have a number of advantages, but there are also disadvantages (Table 1). The combined arthroscopic techniques with the stages of cutaneous suturing and preparation of the tendon increase the risk of infectious complications and do not allow the surgeon to fully maintain the anatomical balance of tension.^10^, ^11^, ^12^ Another potential complication is the perforation of posterior wall and the possible risk of injury n. axillaris, especially when performing subpectoral tenodesis.^13^

**Table 1:** Advantages and Limitations

Advantages
Simplified search for the intertubercular ligament in the subacromial space, marked with a spinal needle from the joint cavity
Optimal visualization of the bicipital sulcus and the tenodesis zone
Maintaining the balance of tension of LHBT by temporary fixed spinal needle or wire below the tenodesis zone
No need to extract the tendon onto the skin surface to prepare for fixation
Stratographic arrangement of neurovascular bundles significantly reduces the risk of damage
Limitations
Severe degeneration and dislocation of the proximal part of LHBT
Severe osteoporosis of proximal humerus

LHBT, long head of the biceps tendon.

The article is devoted to the all-inside arthroscopic method of suprapectoral tenodesis LHBT using the technique of sequential temporary fixation of the tendon with spinal needles (G22) or pins. The needles are used for orientation and for maintaining the anatomical balance of the tendon. The final fixation of the tendon is performed with an interference screw at the border of zones I-II.

## Indications

Indications for tenodesis are as follows: (1) chronic instability of the tendon in the bicipital sulcus; (2) “biceps pulley” lesions; (3) tendinopathy, partial tears of the biceps, tears of the subscapularis tendon; (4) SLAP lesions; (5) ossification of the bicipital sulcus; and (6) calcifying tendonitis.^14^

## Surgical Technique (With Video Illustration)

The operation is performed in the “beach chair” position. The left arm is placed in the position of 30° abduction, 30° flexion, and 10-20° external rotation of the shoulder joint.

**Fig 1:**
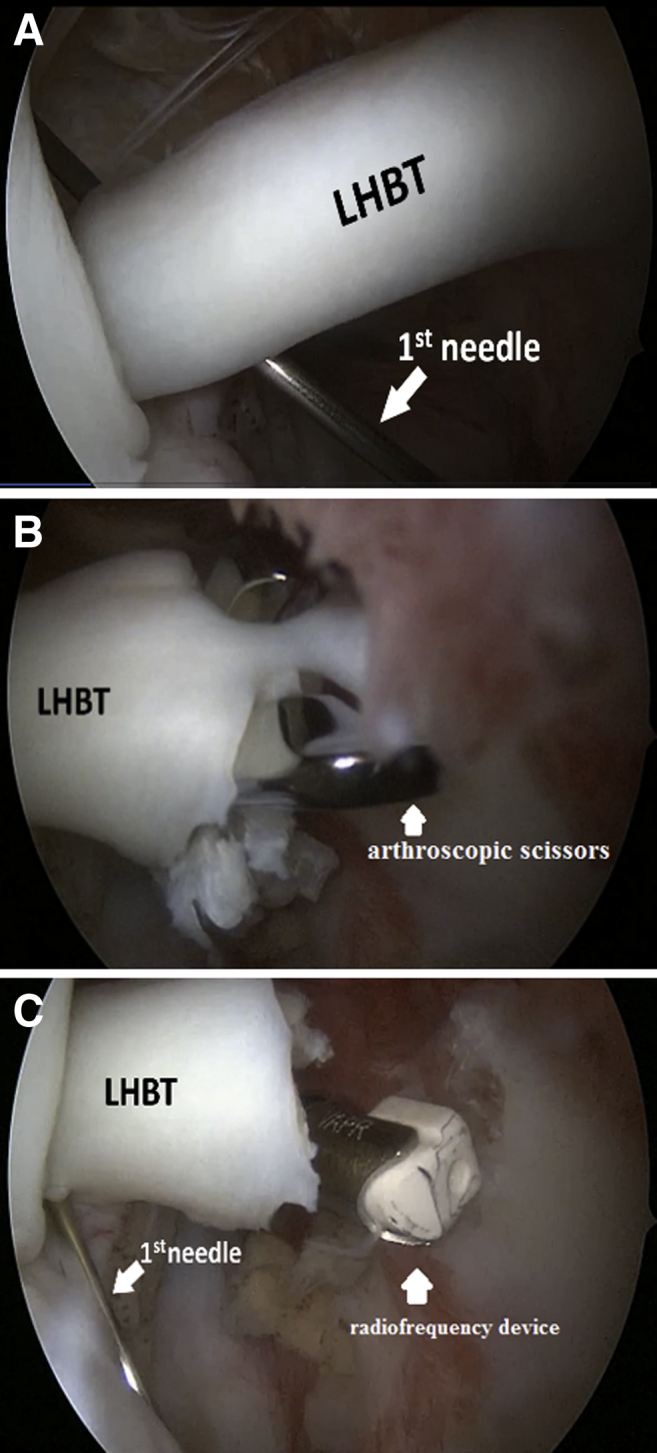
Step 1: Left shoulder is shown in the beach chair position. Arthroscopic view from the posterior portal. (A) Intra-articular LHBT marking-first needle. (B) Tenotomy. (C) Treatment of the stump with the radiofrequency device. (LHBT, long head of the biceps tendon.)

**Fig 2:**
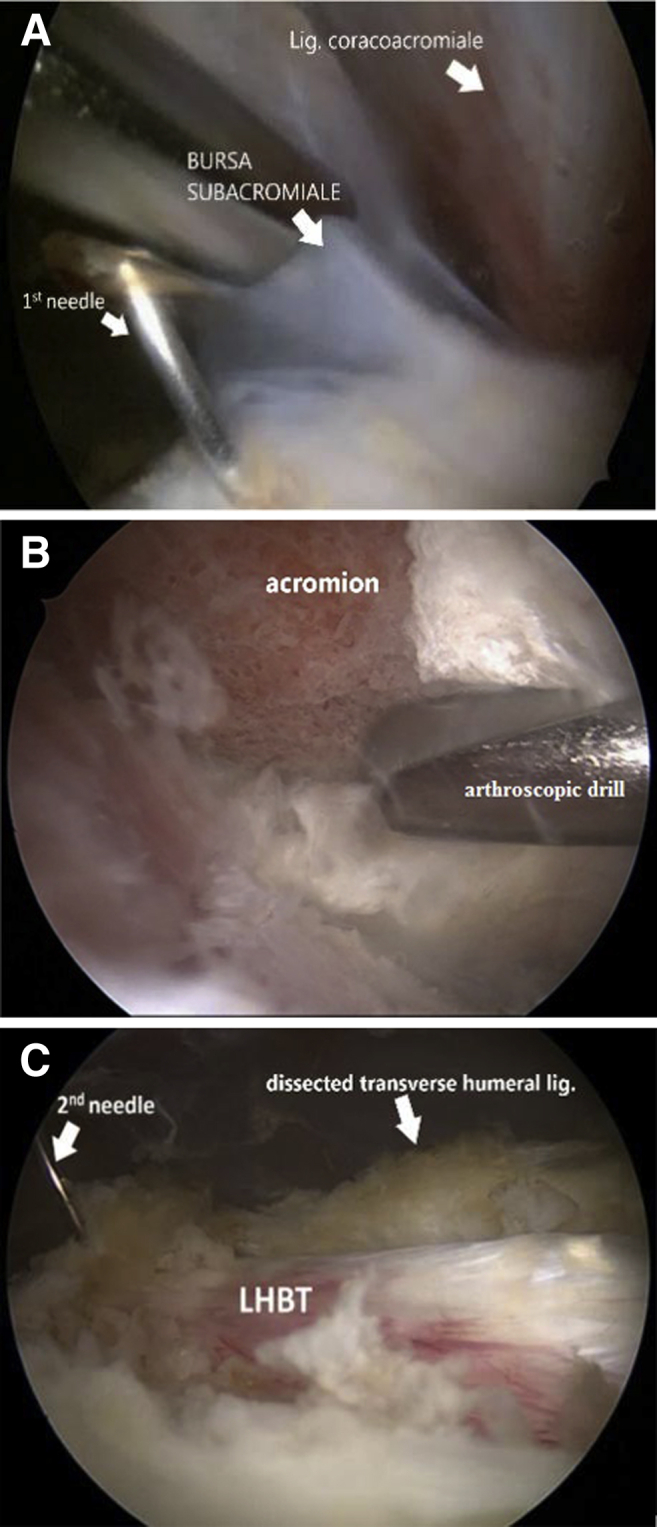
Step 2: Left shoulder is shown in the beach chair position. Arthroscopic view from the anterolateral portal. (A) View the marking needle in the projection of the bicipital groove. Bursectomy, (B) subacromial decompression, and (C) LHBT fixation with the second spinal needle or pin. The transverse ligament is dissected with the radiofrequency device. (LHBT, long head of the biceps tendon.)

The Hopkins II 30° arthroscope (Karl Storz, El Segundo, CA) is inserted into the posterior portal for revision of the intra-articular part of LHBT (Video 1). If there is an indication for tenodesis, the intra-articular part of the biceps is fixed by passing the first spinal needle through the tendon under direct visualization. The next step is tenotomy of LHBT, which is performed close to superior labrum using an arthroscopic scissors or any proper device. The first fixing needle prevents tendon retraction after the tenotomy. Next, a radiofrequency device can be used to prevent further blooding from the stump (Fig 1). After that, the arthroscope is transferred to the subacromial space and bursectomy or acromioplasty is performed as indicated (Fig 2). An anterolateral portal is established and the arthroscope is moved into it.

**Fig 3:**
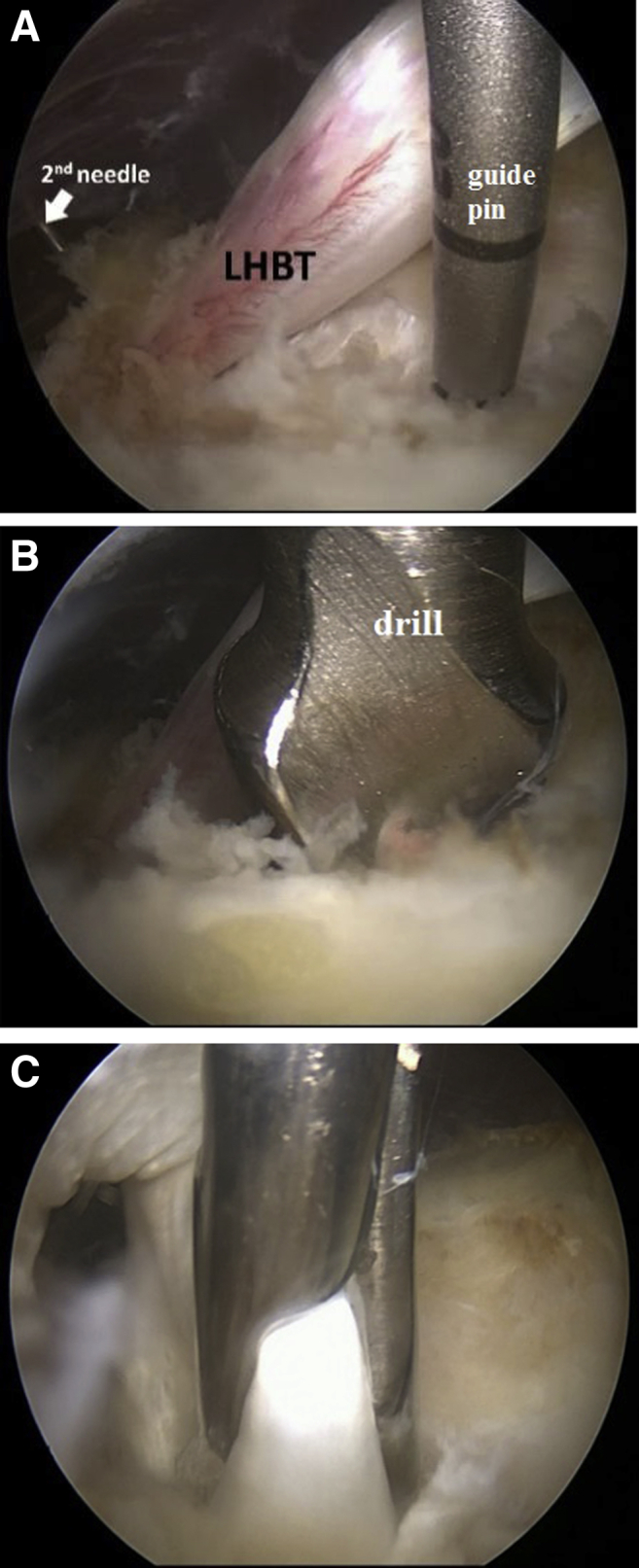
Step 3: Left shoulder is shown in the beach chair position. Arthroscopic view from the anterolateral portal. (A) The tendon is temporarily fixed with the second needle with the humerus. Insertion of the guide pin. (B) Making a hole using a drill bit. (C) Pushing the tendon into the canal with grasper. (LHBT, long head of the biceps tendon.)

**Fig 4:**
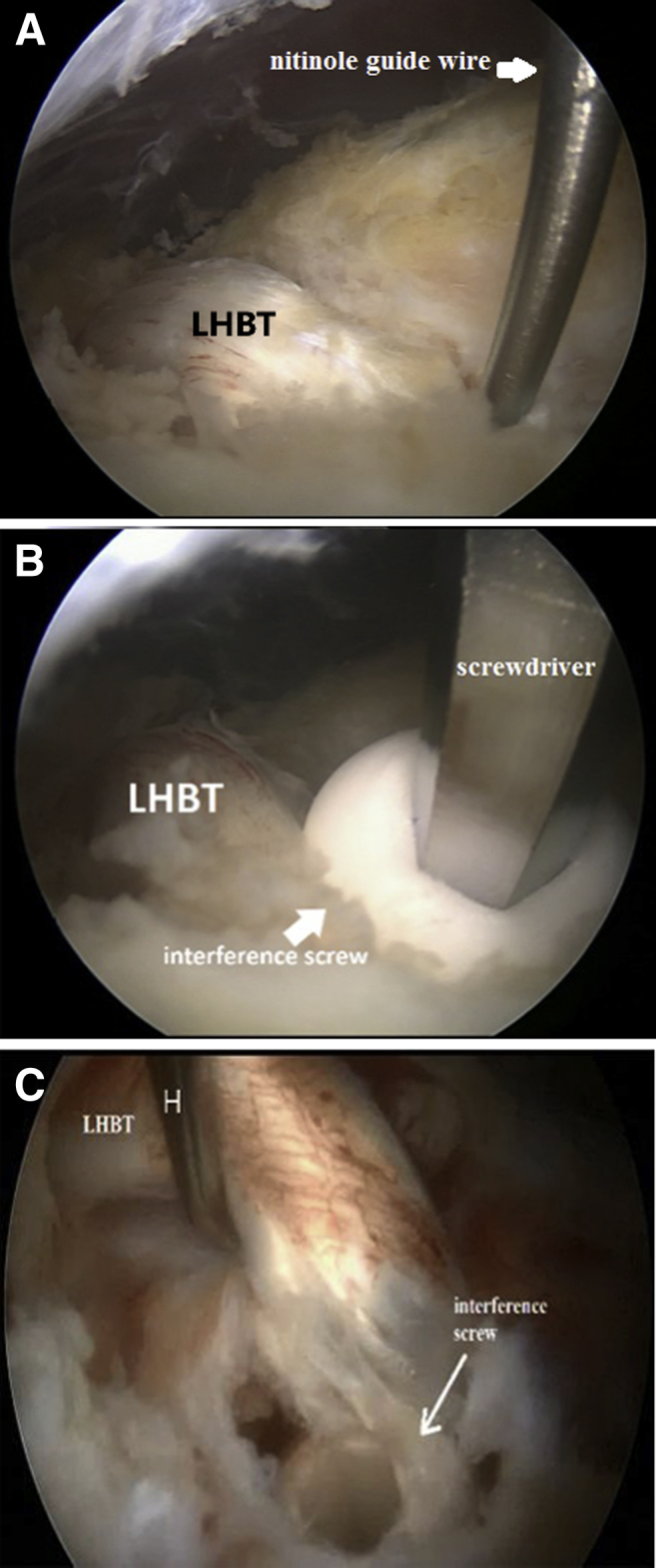
Step 4: Left shoulder is shown in the beach chair position. Arthroscopic view from the anterolateral portal. (A) Inserting the nitinol guide wire into the canal. (B) Inserting the interference screw. (C) Testing the final construct with the hook probe.

The anterolateral portal makes possible to optimally visualize not only the marking needle in the projection of the bicipital sulcus but also n. musculocutaneus in cases of its atypical location.^8^ Next, a third working portal is applied slightly below the marking needle, and the transverse ligament is dissected starting from the spinal needle to the upper edge of the pectoralis major muscle with the radiofrequency device. The second spinal needle or Kirschner pin is used to fix the tendon to the humerus (Fig 3). After fixing the tendon, the first spinal needle is removed. The biceps tendon is removed from the opened groove into the subdeltoid space and the bony bed is treated with a shaver and radiofrequency device to remove the remnants of the inflamed synovial tissue. Then, a blind hole with a diameter corresponding to double diameters of the biceps (usually 7-8 mm) and a depth of 25 mm is drilled in the middle third of the groove along the guide pin. It should be considered that excessive decortication of the bicipital sulcus can lead to bone canal weakening and screw migration. The edges of the canal can be lightly shaved lightly to remove bone chips and sharp parts. At the last stage of the operation, the biceps tendon is inserted into the canal using a grasper or any suitable instrument. The tendon is fixed with an interference screw (Milagro Advance Interference screw, 8 × 23 mm; Mitek Sports Medicine, Chicago, IL) along the nitinol guidewire (DePuy Mitek, Warsaw, IN). After the reliability of fixation is confirmed with the hook probe, the second spinal needle is removed (Fig 4).

## Discussion

LHBT lesions are common causes of shoulder pain and often require surgical treatment. However, there is no consensus on the most advanced surgical technique. Various surgical techniques for tenodesis are discussed but still remain controversial.

Tenodesis is the main surgical option for LHBT problems, and the most important criteria for this procedure are the strength of the fixation and the maintenance of the balance of tension LHBT. This simplified arthroscopic all-inside technique using 2 spinal needles or pins as temporary fixators makes possible to firmly fix the LHBT while maintaining the balance of tension. This technique also significantly reduces the operation time. Optimal visualization minimizes the risk of damage of the regional neurovascular structures, allows high-quality processing and preparation of the bicipital groove for implantation, and the removal of inflamed tenosynovial tissue excludes its nociceptive activity. Table 2 includes tips for the procedure.

**Table 2:** Tips

For temporary fixation of the tendon, we recommend using spinal needles of a suitable diameter.
Alternatively, for distal temporary fixation, a wire can be used instead of spinal needle. This eliminates overtension or weakness of anatomical balance of the tendon.
It is important to avoid excessive decortication of the bicipital groove, which may further cause screw migration.

## Supplementary Data

Video 1 The video describes a technique of arthroscopic suprapectoral tenodesis of the long head of the biceps with interference screw. After diagnostic arthroscopy, the intra-articular part of the biceps is marked by passing the first spinal needle through the tendon. The next step is tenotomy of LHBT, which is performed close to superior labrum using an arthroscopic scissor. LHBT stump treatment is performed with a radiofrequency device. The anterolateral portal visualizes the marking needle in the projection of the bicipital groove. Here bursectomy, subacromial decompression, and LHBT fixation with the second spinal needle or pin are performed. The transverse ligament is dissected with the radiofrequency device. The second spinal needle is used to fix the tendon to the humerus. The biceps tendon is removed from the opened groove into the subdeltoid space. Then, a hole is drilled in the middle third of the groove along the guide pin. In the last stage of the operation, the biceps tendon is inserted into the canal using a grasper. Along the nitinol guidewire in the canal, the tendon is fixed with an interference screw. After the reliability of fixation is confirmed with the hook probe, the second spinal needle is removed. (LHBT, long head of the biceps tendon.)

ICMJE author disclosure forms
